# Shifting practice: Moving to a stent first approach for both left and right sided acute malignant large bowel obstruction (LBO)

**DOI:** 10.1007/s00464-025-12035-2

**Published:** 2025-08-26

**Authors:** Emily Farrow, Shona Gardner, Neil Collin, Anne Pullyblank

**Affiliations:** 1 North Bristol NHS Trust, Bristol, UK; 2 School of Medicine, Cardiff University, Cardiff, UK

**Keywords:** Colonic stenting, Large bowel obstruction

## Abstract

**Background:**

To assess the safety and efficacy of a change to a stent first approach for malignant large bowel obstruction (LBO) in both left and right colon in a single centre over a 4-year period.

**Methods:**

This retrospective cohort study in an acute NHS Hospital Trust from 01/01/2019–31/12/2022 examines a change in practice from emergency surgery (ES) to colonic stenting for patients with both left and right sided acute malignant LBO. Co-primary outcomes were clinically successful bowel decompression following stenting and 30-day mortality. Secondary outcomes were length of stay, stent complications, stoma formation and minimally invasive surgery (MIS).

**Results:**

68 patients underwent colonic stenting, and 29 patients underwent primary ES for acute malignant LBO. Stenting achieved successful bowel decompression in 77.9%. 30-day mortality for those initially stented was 7.4% and for ES 6.9%.

In palliative patients initially treated with stenting the stoma rate was lower (15.4 vs. 100.0%) with a reduced rate of open surgery (5.1 vs 87.5%) when compared to ES. In curative patients initially treated with stenting the stoma rate was lower (37.9 vs. 80.1%) with an increased rate of MIS (69.0 vs 19.0%), when compared to ES. 27.9% of patients underwent stenting proximal to the splenic flexure.

**Conclusions:**

It is possible to offer colonic stenting to > 80% of patients presenting with acute malignant LBO despite not having a 24/7 rota. There was a reduced rate of stoma formation, open surgery and length of stay in both palliative and curative patients undergoing primary colonic stenting.

**Graphical abstract:**

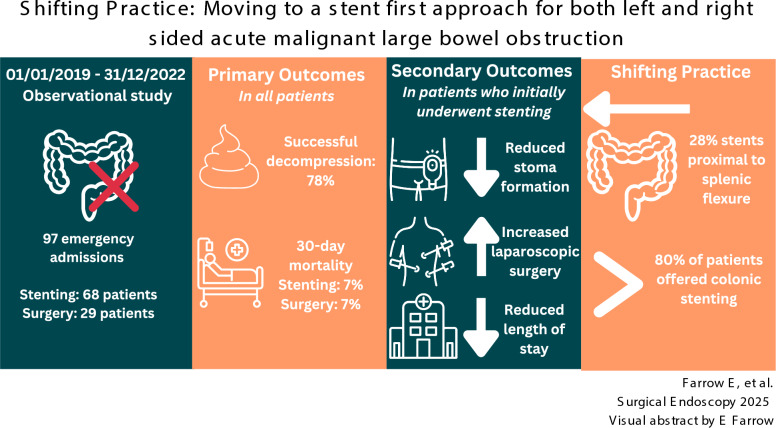

Colorectal cancer remains the fourth most common cancer in the UK with 44100 new cases per year [1]. A national screening programme for colorectal cancer was introduced in 2006, but despite a 70% uptake, approximately 15% of colorectal cancer presents as an emergency [2, 3]. Nearly 10% of all emergency laparotomies in the UK are performed for colorectal cancer and 80% of these are for malignant large bowel obstruction (LBO) [4, 5].

Colonic stenting was initially recommended only for palliation of malignant LBO [6]. This was based on two randomised trials that closed prematurely because of adverse outcomes in the colonic stenting group [7, 8]. In 2020 European Society of Gastroenterology guidance was updated to include colonic stenting as an option in potentially curable left-sided obstructing colon cancer based on high-quality evidence from CReST and ESCO trials which showed no difference in 3-year overall survival and disease-free survival rates [9–11]. The CreST trial also demonstrated a significant reduction in long-term stoma formation when stenting was used as a bridge to surgery compared to emergency surgery (ES) [10].

Most studies have focused on left-sided tumours with the end point being reduction in stoma formation. However, 32.5–54.0% of emergency surgeries for colonic obstruction are performed for tumours in the proximal colon [12, 13]. As expertise has developed, stenting has become an option for tumours at all sites except the rectum and caecum. Although resection of right-sided tumours is less likely to require a stoma, there are perceived benefits to converting an emergency operation in a physiologically unwell patient to an elective operation which is more likely to be minimally invasive. Minimally invasive surgery for colorectal cancer is shown to result in better surgical and patient-reported outcomes compared with open surgery [14–16].Although laparoscopic resection for urgent colorectal cancer resection has increased, laparoscopic surgery only occurs in 30% of cases with an almost 20% conversion rate [17].

There are both clinical and logistical challenges to offering patients colonic stenting in the emergency setting. Clinical contra-indications include impending or confirmed bowel perforation and tumour location e.g., caecum. Logistical challenges include a lack of local expertise and out of hours availability of both surgeon and interventional radiologist. A UK cross-sectional study of provision of colonic stenting for left-sided tumours showed that only 1 in 4 hospitals have access to stenting out of hours and at weekends [18]. The recent NBOCA State of the Nation report shows that the number of patients having major resection during an emergency admission varies from 3 to 24%. Only 15.7% of hospitals having a major resection rate of < 10%, reflecting variation in access to colonic stenting [17]. Despite not having a 24/7 rota for stenting, we have adopted a proactive approach to offering a stent in patients with malignant LBO in both the left and right colon.

The aim of this study is to assess safety and efficacy of a change in practice from emergency surgery to colonic stenting in patients with malignant LBO in both left and right colon in a single centre over a 4-year period.

## Methods

All adult patients presenting with large bowel obstruction considered due to colonic malignancy on imaging to an 800-bed acute NHS Hospital Trust in the UK between 1 January 2019 and 31 December 2022 were included. Patients were fit enough to undergo emergency intervention, either stenting or surgery. Data were collected on the National Emergency Laparotomy Audit (NELA) database for patients undergoing emergency surgery (ES) and radiology reporting systems for colonic stenting. This data were retrospectively reviewed and supplemented by clinical notes.

Colonic stenting was performed by a colorectal surgeon and interventional radiologist in the radiology department under combined endoscopic and fluoroscopic guidance. Stenting was routinely available Monday to Friday 08:00–18:00. Out-of-hours and at weekends, it depended upon expertise available. 1-in-2 weekends were covered by a colorectal surgeon, so colonic stenting would be available on these weekends.

This unit had taken part in the CreST trial which stipulated that units were required to have performed 30 stents for obstructing colorectal cancer, and any participating radiologist must have undertaken at least 10 stents prior to participation [10]. Currently 90% of stents placed to relieve obstruction are uncovered i.e., the stents are made of bare metal. The alternative is a stent with a plastic covering designed to reduce the risk of tumour growing into the lumen and causing re-obstruction [19]. Between June 2017 and April 2023, i.e., during our study period, we participated in the CreST2 trial, comparing uncovered vs covered stents (EGIS, BVM Medical Ltd) and so patients were randomised to either type of stent during this period. There was no antibiotic prophylaxis. Sedation with midazolam and pain relief with fentanyl was provided for more proximal tumours or at patient request. Stenting was performed with standard monitoring.

Co-primary outcomes were clinically successful bowel decompression in the colonic stenting group and 30-day mortality. Secondary outcomes were length of hospital stay, stent complications and rate of stoma formation.

Stent complications include failure to deploy and therefore failure to decompress the bowel, bowel perforation, stent migration and re-obstruction. Stent-related complications were classified as immediate (< 24 h), early (1–7 days) or late (> 7 days). Patients who failed stenting underwent appropriate ES; outcomes were included in intention to treat analysis.

## Results

Between 1 January 2019 and 31 December 2022, 73 patients underwent colonic stenting, and 29 patients underwent primary emergency surgery (ES) for acute malignant LBO. Of the 73 that were stented, 5 were excluded as they presented with re-obstruction of a previous stent, leaving 68 stented patients in our study population. Median age was 73 years (mean 70; range 36–98), 50.0% were male. During the same period, 29 patients underwent ES; the median age was 68 years (mean 60; range 23–91), 44.8% were male. 72.4% (21/29) of emergency laparotomies were performed with curative intent (Table 1).

**Table 1:** Baseline characteristics

All Patients (Curative & Palliative)		
	Colonic Stenting (*n* = 68)	Emergency Surgery (*n* = 29)
Age (years)		
Median	72.7	68.9
Mean	70.2	66.3
Range	36–98	23–91
Sex		
Male	34	13
Female	34	16
Tumour site		
Rectosigmoid	33	10
Descending	6	0
Splenic	10	4
Transverse	8	6
Hepatic flexure	7	4
Ascending	4	4
Caecal	0	1
Total proximal	27.9% (19/68)	51.2% (15/29)
Treatment intent		
Curative	42.6% (29/68)	72.4% (21/29)
Palliative	57.4% (39/68)	37.6%% (8/29)

**Fig. 1:**
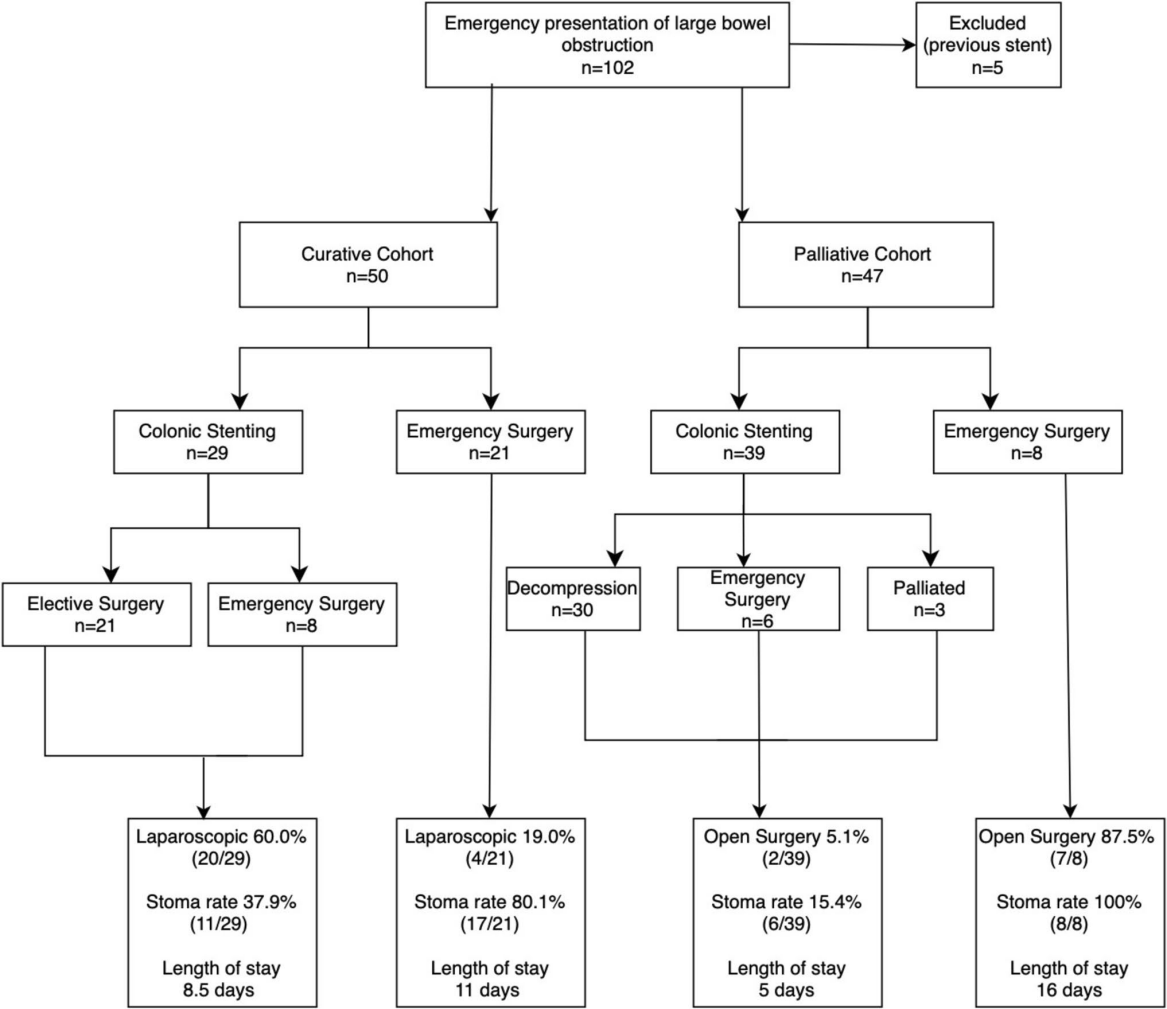
A flow chart showing the treatment pathway and outcomes for all patients presenting with acute malignant LBO

Of the 68 patients that initially underwent colonic stenting, 42.6% (29/68) were with curative intent and 57.4% (39/68) palliative (Fig. 1). Successful bowel decompression at the time of stenting was achieved in 77.9% (53/68 patients). 30-day mortality was 7.4% (5/68 patients). 75.0% (51/68) of patients had a new diagnosis of bowel cancer at the time of presentation and 25.0% (17/68) of patients had a pre-existing diagnosis of bowel cancer. 27.9% (19/68) of colonic stents were placed in the right colon i.e., proximal to the splenic flexure. The proportion of patients offered colonic stenting rather than ES, increased from 57.1% (12/21) in 2019 to 83.4% (15/18) in 2022, the proportion of proximal stents increased from 16.7% in 2019 to 33.3% in 2022 (Table 5). In 2019/20, 72% of patients were treated with curative intent whereas in 2021/22, this dropped to 31%.

To compare stenting vs emergency resection, we have analysed data for curative and palliative cohorts separately as stenting was a definitive procedure in palliative patients.

### Curative cohort

On an intention to treat basis, 79.3% (23/29) patients that underwent colonic stenting with curative intent had clinically successful bowel decompression with 3.4% (1/29) 30-day mortality rate. 72.4% (21/29) of patients subsequently underwent an elective resection. 85.7% (18/21) were performed laparoscopically, 14.3% (3/21) with a stoma. Median time to elective surgery was 37 days, (mean 46 days, range 19–214 days). One patient underwent neoadjuvant chemotherapy for locally advanced disease which meant a delay of 214 days to their elective resection. All patients had R0 resections, there were no anastomotic leaks and 0.0% 30-day mortality. The median length of hospital-stay for stenting and elective resection combined was 8 days (mean 11 days, range 3–38 days), divided into median 4 days post stenting and 4 days post elective resection.

Of the 21 patients undergoing primary ES with curative intent the 30-day mortality was 9.5% (2/21). 80.1% (17/21) had resectional surgery with stoma formation. 19.0% (4/21) underwent minimally invasive surgery. Median length of stay was 11 days (mean 17 days; range 7 to 49 days) (Table 3). 40.9% (9/21) had CT evidence of perforation, impending perforation or intussusception and therefore went straight to surgery. 4.8% (1/21) were thought to have diverticular disease on imaging.

The rate of stent complications was 27.6% (8/29), 4 failed due to inability to pass a guidewire, 2 perforated and there were 2 re-obstructions at 20 and 125 days (Table 2). The overall rate of stoma formation, including both elective resections and ES following failed stenting was, was 37.9% (11/29). The overall median length of stay was 8.5 days. The overall rate of minimally invasive surgery was 69.0% (20/29) (Table 3).

**Table 2:** Stent complications

	Immediate < 24H	Early 1–7 days	Late > 7 days
**Curative**			
Failure to deploy the stent	4/29		
Perforation		1/29	1/29
Re-obstruction			2/29
**Palliative**			
Failure to deploy the stent	7/39		
Perforation		1/39	1/39
Re-obstruction			3/39
Total	16.1% (11/68)	2.9% (2/68)	10.3% (7/68)

**Table 3:** Comparison of primary & secondary outcomes curative and palliative cohorts

Curative Patients		
	Stenting	Emergency Surgery
Successful bowel decompression	79.3%	N/A
30-day mortality	Successfully stented: 0.0% Failed stenting: 3.4%	9.5%
Stent complications	27.6%	N/A
Rate of stoma formation	37.9%	81.8%
Median length of stay (days)	8.5	15
Minimally invasive surgery	69.0%	19.0%
Palliative patients		
	Stenting	Emergency Surgery
Successful bowel decompression	77.0%	N/A
30-day mortality	Successfully stented: 2.6% Failed stenting: 7.7%	0.0%
Stent complications	30.8%	N/A
Rate of stoma formation	15.4%	100.0%
Median length of stay (days)	8	16
Minimally invasive surgery Open surgery	10.3% 5.1%	12.5% 87.5%

### Palliative cohort

Of 39 colonic stents placed for palliation; 69.2% (27/39 patients) had metastatic disease and 30.8% (12/39 patients) were deemed unfit for ES. Successful bowel decompression was achieved in 77.0% (30/39). 7 (17.9%) failed due to inability to get a guidewire across the obstructing lesion and 2 (5.1%) perforated. 3 patients (7.7%) re-obstructed after > 1 year (Table 2). Of the 12 patients with stent complications, 6 underwent ES with stoma formation, 4 with MIS.

On an intention to treat basis, 77.0% (30/39) patients that underwent colonic stenting with palliative intent had a clinically successful bowel decompression with a 10.3% (4/39) 30-day mortality rate. The rate of stent complications was 30.8% (12/39), which includes late re-obstructions. The rate of stoma formation was 15.4% (6/39). The median length of stay was 5 days (mean 9, range 1–41). The rate of open surgery was 5.1% (2/39) (Table 3).

Of the 8 undergoing ES with palliative intent, 100.0% (8/8) had a stoma formed 87.5% (7/8) with open surgery. Median length of stay was 16 days (mean 20 days; range 8–42 days). There was 0% 30-day mortality.

### Proximal stents

27.9% (19/68) of colonic stents in this study were placed proximal to the splenic flexure i.e., transverse colon, hepatic flexure, or ascending colon, 47.4% (9/19) with curative intent. Successful bowel decompression was achieved in 73.7% (14/19) and 30-day mortality was 15.8% (3/19). 6/19 had ES after failed colonic stenting or complications, 5/19 went onto have elective surgery and 8/19 had no further intervention (Table 4).

**Table 4:** Comparison of proximal and distal colonic stents

	Proximal Colonic Stenting	Distal Colonic Stenting	*P*-value (< 0.05)	Proximal Emergency Surgery
Proportion of curative intent	47.4% (9/19)	40.8% (20/49)	0.69	14/17
Successful bowel decompression	73.7% (14/19)	79.6% (39/49)	0.82	N/A
30-day mortality	15.8% (3/19)	4.1% (2/49)	0.16	11.7% (2/17)
Stent complications (perforation/ failure)	26.3% (5/19)	20.4% (10/49)	0.63	N/A
Stent complication (re-obstruction)	10.5% (2/19)	6.1% (3/49)	0.56	N/A
Rate of stoma formation	36.8% (7/19)	20.4% (10/49)	0.24	76.5% (13/17)
Median length of stay (days)	7	5		16
Emergency surgery	36.8% (7/19)	18.4% (9/49)	0.19	14/17 Open
Elective surgery	26.3% (5/19)	32.6% (16/49)	0.70	

## Discussion

The overall rate of clinically successful bowel decompression was 77.9%, which is comparable to existing trial data [10, 20]. Of note, two stent failures were in patients with benign pathology, not recognised on pre-operative CT. The 30-day mortality on an intention to treat basis for patients undergoing colonic stenting was 7.4% compared with 6.9% undergoing emergency surgery (ES). The overall stent failure rate was 16.1% with a 2.9% early perforation rate and 2.9% late (> 7 days) perforation rate.

Practice evolved over the study period as the team became more experienced, with more stents being offered and more proximal stents being performed later in the series (Table 5). By 2022, 83.4% of patients were offered colonic stenting, 33.3% proximal to the splenic flexure.

**Table 5:** Change in practice over time

	Curative Stent	Curative Laparotomy	Palliative Stent	Palliative Laparotomy	Proportion of Stented Patients	Proximal Stent	Distal Stent
2019	5	9	7	0	57.1%	2 (16.7%)	10
2020	16	6	5	2	72.4%	6 (28.6%)	15
2021	6	5	14	5	66.7%	6 (30.0%)	14
2022	2	2	13	1	83.3%	5 (33.3%)	10
Total	29	22	39	8	70.1%	19	49

Of a total of 97 patients, 15 were not clinically appropriate for colonic stenting (12 had evidence of perforation on CT scan, 2 were initially thought to have benign disease and 1 patient had a caecal tumour). Therefore, 82.9% (68/82) of clinically appropriate patients presenting with acute malignant LBO initially underwent colonic stenting, despite not having a 24/7 colonic stenting service. Most patients (80.9%) were safely stented within 48 h of diagnosis. No stents were performed out of hours.

A total of 17.1% (14/82) of patients who had ES (both curative and palliative cohorts) might have been clinically appropriate for stenting when examined retrospectively. Some were probably not stented due to organisational factors (e.g., 3/14 underwent ES on a non-colorectal weekend). However, 9/14 presented in 2019 and 8/14 patients had disease proximal to the splenic flexure so this was probably due to less established practice earlier in our study period.

It is likely that the Covid-19 pandemic influenced clinical presentation during the study period as 69% of procedures were with palliative intent in 2021/22 versus 28% in 2019/20. This is in line with concerns that post pandemic patients tended to present later with more advanced disease [21]. In addition, 72.7% of patients with a previously diagnosed cancer presented as an emergency in 2021/22 compared with 27.3% in 2019/20. This may reflect delays in treatment because of the Covid-19 pandemic backlog [22].

### Curative cohort

To draw meaningful comparisons regarding outcomes such as stoma rate, mortality and rate of minimally invasive surgery it is necessary to compare just patients treated with curative or palliative intent (Table 4). The 30-day mortality is lower in patients initially treated with colonic stenting compared with ES (3.4 vs. 9.5%). The stoma rate was also markedly reduced (37.9 vs. 80.1%) when compared to ES. This is a more marked difference than CREST trial which found that colonic stenting reduced stoma rates by 20%, but a comparable figure to a 2020 meta-analysis of 2839 patients [10, 23]. The majority (72.7%) of stomas were formed for patients in whom colonic stenting failed/ perforated and then required ES.

The rate of minimally invasive surgery was also markedly increased (19.0 vs. 69.0%) for patients undergoing colonic stenting compared to ES. This is reflected in other studies [24].The overall median length of stay in patients undergoing stenting followed by elective surgery was 8 days, compared to 11 days in those undergoing ES. CREST included duration of hospital stays within the first year after randomisation rather than just the events related to surgery and found no significant difference in duration of hospital stay in patients undergoing stenting versus ES [10].

Our patients undergoing colonic stenting with curative intent had a lower mortality, reduced rate of stoma formation, higher rate of minimally invasive surgery and reduced length of stay compared with those patients that initially underwent ES.

### Palliative cohort

ESGE recommends colonic stenting for palliation of malignant colonic obstruction [9]. Studies have confirmed that colonic stenting is associated with significantly shorter hospitalisation, which allows patients to progress to chemotherapy more rapidly [25]. This is reflected in our cohort with median length of stay was 5 days in patients undergoing colonic stenting, compared to 16 days in those undergoing ES, likely due to a longer recovery period, stoma training and complications. The rate of stoma formation in patients undergoing colonic stenting with palliative intent was 15.4%, which is similar to data from meta-analyses (stoma rate 12.7–14.3%) [25, 26].

The 30-day mortality rate was 10.3% for those undergoing stenting versus 0.0% in those undergoing ES but meta-analysis data which shows that there is no significant difference in 30-day mortality between stenting and ES [26]. 30.8% (12/39) of patients that went straight to palliative stenting were considered unfit to have ES and without stenting would have likely died of acute LBO during admission. Following colonic stenting these patents went onto live on average for > 1 year (median 496 days, mean 543, range 5–1159 days).

### Proximal stenting

There is a paucity of data on the role of colonic stenting proximal to the splenic flexure. In the CREST trial only 3.3% of stents were placed proximal to the splenic flexure, even when splenic flexure tumours are included this only accounts for 9.0% of tumours [10].

Some studies have indicated promising results with proximal stenting. In 2017 a multi-centre trial of proximal colonic stenting in the palliative setting included 69 patients with clinical relief achieved in 78% of these patients [27]. In 2020 a Japanese national database study compared outcomes in patients undergoing emergency colectomy versus colonic stenting as a bridge to surgery with right-sided colonic obstruction [28]. 1500 pairs of patients were generated through propensity matching; they demonstrated reduced stoma rate, reduced morbidity and reduced hospital stay in those patients initially undergoing stenting. However, this study did not report the rate of successful bowel decompression with stenting or stent complications.

We describe a lower rate of successful bowel decompression, higher rate of stoma formation, stent complication and increased length of stay when compared to distal stenting but the numbers are small and not statistically significant. However, when compared to emergency right hemi-colectomy, proximal stenting has a lower rate of stoma formation (36.8 vs 76.5%) and reduced length of stay (7 vs.16 days).

## Conclusion

There is a large national variation in stenting. 2023 NBOCA data show the average number of patients having emergency resection nationally is 15% compared to 6% in our trust. In 2023 only 1.6% of emergency patients were stented, dropping to 1.3% in 2024 [17]. Our study shows that it is possible to offer colonic stenting to a high proportion (> 80%) of patients presenting with acute malignant LBO, despite not having a 24/7 rota and most patients can safely wait 48 h.

There was a reduced rate of stoma formation, open surgery and length of stay in both palliative and curative patients undergoing colonic stenting prior to surgical intervention.

We also offered colonic stenting to more patients presenting with proximal obstruction than has previously been reported in the literature. This was associated with a higher risk of unsuccessful bowel decompression, stent complication and stoma formation when compared to distal obstruction but still had some advantages when compared with ES for proximal obstruction.
